# Disability-Adjusted Life Years (DALYs) Due to Ischemic Heart Disease (IHD) Associated with Natural Disasters: A Worldwide Population-Based Ecological Study

**DOI:** 10.5334/gh.919

**Published:** 2021-04-28

**Authors:** Kai-Sen Huang, Debarati Guha-Sapir, Qian-Lan Tao, Yan-Yan Wang, Xiao-Jian Deng, Tao Xiao, Yong-Qiang Yang, Ding-Xiu He

**Affiliations:** 1 Department of Cardiology, People’s Hospital of Deyang City, Affiliated Hospital of Chengdu Medical College, Deyang, Sichuan, CN; 2 Department of Cardiology, The Affiliated Hospital of Southwest Medical University, Luzhou, Sichuan, CN; 3 The World Health Organization Collaborating Center for Research on the Epidemiology of Disasters, Institute of Health and Society, University of Louvain, Brussels, BE; 4 Center of Gerontology and Geriatrics & National Clinical Research Center for Geriatrics, West China Hospital, Sichuan University, Chengdu, CN; 5 Department of Respiratory and Critical Care Medicine, West China Hospital, Sichuan University, Chengdu, CN; 6 Department of Emergency, People’s Hospital of Deyang City, Affiliated Hospital of Chengdu Medical College, Deyang, Sichuan, CN

**Keywords:** natural disaster, disability-adjusted life years, ischemic heart disease, ecological study

## Abstract

**Background:**

Recent studies have reported an association between natural disasters of various kinds and ischemic heart disease (IHD). We investigated the association between Disability-adjusted life years (DALYs) due to IHD and natural disasters and aimed to assess DALYs as a quantification of the burden of IHD related to natural disasters at the global level.

**Methods:**

Country-specific data of natural disaster impacts DALYs due to IHD and socioeconomic variables were obtained from open sources over the period of 1990–2013 and 2014–2017. A population-based trend ecological design was conducted to estimate the association between trends in DALYs and natural disasters (occurrence, casualties and total damage), adjusting for socioeconomic variables.

**Results:**

Most countries have experienced increases in natural disaster occurrences and decreases in DALYs during this study period. The unadjusted correlation analysis demonstrated a positive and significant correlation between DALYs and natural disasters for females and for both sexes (R = 0.163 and 0.146, p = 0.024 and 0.043), and a marginally significant correlation for males (R = 0.128, p = 0.076). After adjusting for socioeconomic variables, multiple linear regression demonstrated independent associations between the occurrence and DALYs due to IHD for males, females and both sexes (standardized coefficients = 0.192, 0.23 and 0.187, p = 0.016, 0.004 and 0.022).

**Conclusions:**

A weak but significantly positive association between natural disaster and IHD was confirmed and quantified at the global level by this DALY metric analysis. Adaptation strategies for natural disaster responses and IHD disease burden reduction need to be developed.

## Introduction

Ischemic heart disease, characterized by an inadequate blood supply to the heart, is among the top contributors to DALYs, a common metric originally developed by World Health Organization (WHO) to assess the global disease burden. Over the past 30 years, the number of DALYs due to IHD have increased from 119,479,835.80 in 1990 to 170,275,348.13 in 2017. Globally, IHD is one of the five leading causes of DALYs and accounted for 6.83% of global DALYs in 2017 [1,2].

Recently, the association between natural disasters and IHD has been revealed by numerous epidemiological and clinical studies [3,4]. A natural disaster is defined by the World Health Organization as an act of nature of such magnitude as to create a catastrophic situation, which has an immediate impact on the population and often results in the destruction of the physical, biological and social environment of those affected, creating a large public health problem. In the past few decades, an accumulation of evidence points towards adverse impacts of natural disaster exposure on IHD outcomes, with the underlying mechanisms of this phenomenon including environmental contamination exposure, emotional stress and lifestyle changes [5,6].

As a result, a detailed analysis and description is needed of the adverse impact of different kinds of natural disasters on the disease burden due to IHD to promote a better understanding and preparedness for natural disasters. Fortunately, the Institute for Health Metrics and Evaluation (IHME) performed a comprehensive systematic review and calculated the Global Burden of Disease (the Global Burden of Disease Study, GBD) from 1990 to 2017 [2]. Combined with data on natural disasters from the Centre for Research on the Epidemiology of Disasters (CRED) [7], such data offered us an opportunity to carry out an ecological study using a longitudinal approach with a repeated measure analysis. The aim of our study was to assess DALYs as a quantification of the burden of IHD related to natural disasters in 193 countries from 1990 to 2017, to promote appropriate adaptation actions and strategy development for public health.

## Methods

### Study design

We conducted an ecological study of trends in annual means for natural disasters impacts and confounding factors, and for DALYs rate for the period 1990 to 2017 in 193 countries, with the whole population involved. The annual means of each variable were calculated with original data from two periods (1990–2013 and 2014–2017). The country-level trends in variables for the two periods can be calculated based on the following formula:

Trend in variable_a_ = mean of variable_a_ (2014–2017) – mean of variable_a_ (1990–2013).

All of the original data for every variable were collected from the same 193 countries. Countries with any unavailable data on natural disasters or DALYs were excluded. Our study is a population-based ecological study using data freely accessible from open sources with no identification of individuals, rendering informed consent from participants or approval from the Ethics Research Committee waived.

### Data sources and definitions

The analysis of trends in variables would be possible with original data from two periods, 1990–2013 and 2014–2017, which were freely obtained from open sources, including: CRED, GBD, the World Bank and the Food and Agriculture Organization of the United Nations (FAO).

#### Independent variables—Impact of natural disaster

Impact of natural disasters were quantified as the occurrence, deaths and total affected, which were obtained from the Emergency Events Database (EM-DAT) in CRED [7]—a WHO Collaborating Centre since 1980. EM-DAT contains essential core data on the annual occurrence and effects of natural and technological disasters around the world from 1900 to the present. A natural disaster includes all kinds of main categories of catastrophic events of nature, including geophysical, meteorological, hydrological, climatological, biological and extra-terrestrial disasters. For a disaster to be entered into the EM-DAT database, at least one of the following criteria must be fulfilled: (1) Ten or more people are reported killed. (2) One hundred or more people are reported affected. (3) Declaration of a state of emergency. (4) Call for international assistance. The variables of natural disaster exposure were defined as follows:

Occurrence (per year): The average annual number of natural disasters in each country in a certain period.

Casualty (per 100,000 per year): The average annual number of deaths, injured, homeless, and affected people requiring immediate assistance during a natural disaster in a certain a period, i.e., requiring basic survival needs such as food, water, shelter, sanitation and immediate medical assistance.

Total damage (1,000 USD per year): The value of all damages and economic losses directly or indirectly related to the disaster. This information may include the breakdown figures by sectors: Social, Infrastructure, Production, Environment and Other (when available).

#### Independent variables—confounding variables

Another 10 variables were collected as confounding factors from two sources: The World Bank and the Food and Agriculture Organization of the United Nations (FAO). Seven variables came from the World Bank [8], including: life expectancy (years, for males, females and both sexes), GDP per capita (constant 2010 USD), industry (value added, constant 2010 USD), government expenditure on education (% of GDP), CO_2_ emissions (metric tons per capita) and urban population (%), trade (% of GDP). Additionally, the total population of each country was obtained from the World Bank as a weight factor in multivariate regression. Three variables came from FAO [9], including: tobacco consumption per capita (kg), alcohol consumption per capita (kg), fat and meat consumption per capita (kg).

#### Dependent variables—DALYs due to IHD

DALYs due to IHD were assessed according to the International Classification of Diseases (most recently ICD-10 I20–I25 and ICD-9 410–414). The data on DALYs were collected from the GBD. The GBD provides the data of DALYs due to IHD from 1990 to 2017 for males, females and both sexes separately [2]. We used the DALYs definition as annual DALYs due to IHD per 100,000 population of interest (all ages) in that year.

### Statistical analysis

Statistical analyses were conducted on 193 countries using IBM SPSS statistical program version 22 (IBM, Armonk, New York, USA), and a P value < 0.05 was considered to be statistically significant. Continuous variables are presented as mean ± SD and categorical variables are presented as percentages.

A one-sample T-test was used to assess whether the trends in natural disaster impact and DALYs rates were statistically different from the test value of zero. The bivariate Pearson correlation test was used as the measure of associations between trends in natural disaster impact and DALYs rates. The correlations were visualized by a world map of trends with Quantum Geographic Information Systems (QGIS) (OSGeo, Beaverton, OR, USA).

Multivariate linear regression was used to investigate the association between trends in dependent variables and independent variables. Dependent variables included trends in DALYs due to IHD for males, females and both sexes. Independent variables included trends in natural disaster impact (occurrence, casualty and total damage) and confounding variables (life expectancy, GDP per capita, industry, government expenditure on education, CO_2_ emissions, urban population, trade, tobacco, alcohol, fat and meat consumption). Multivariate linear regression models were conducted with stepwise methodology, weighted by the average population from 1990–2017. Coefficients with the 95% confidence intervals (95% CI) and p value are reported for variables included in the multivariate linear regression. Additionally, standardized coefficients were calculated to evaluate the size of the effect for all variables. All of the analyses were performed separately for males, females and both sexes.

## Results

**Figure 1:**
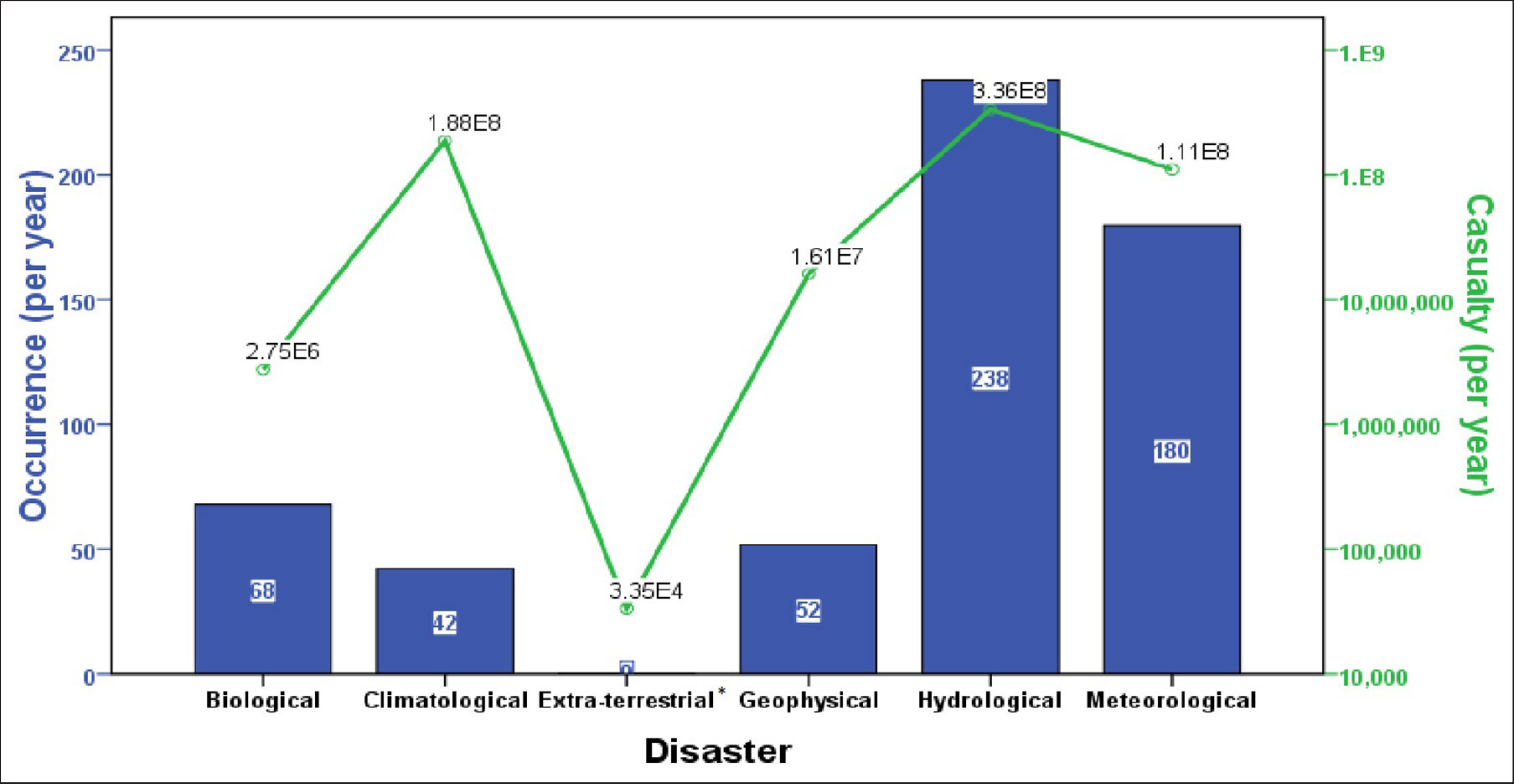
The average occurrence and casualty for subgroups of natural disasters from 1990 to 2017. * The average occurrence for extra-terrestrial disasters is 0.056.

**Figure 2:**
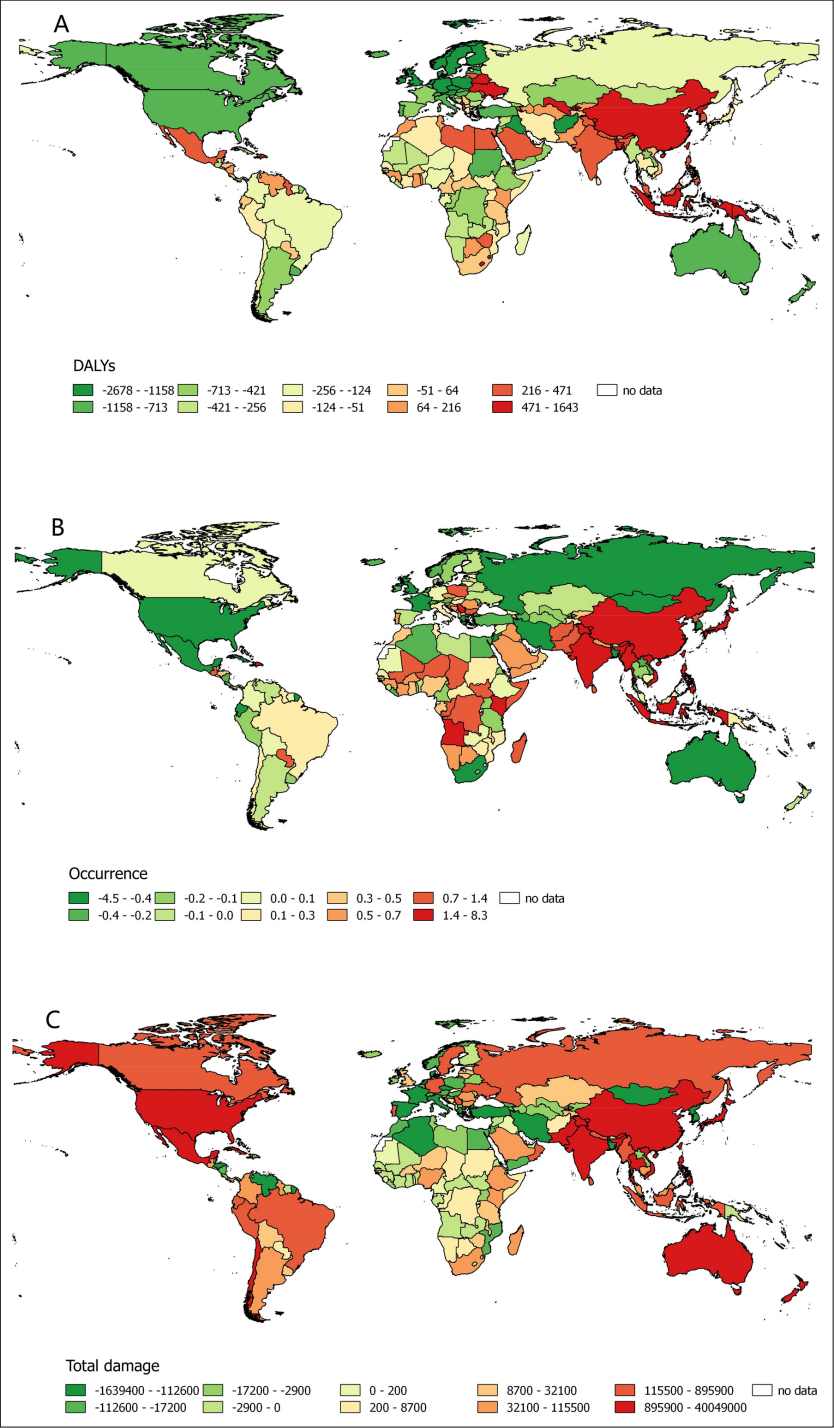
Map of trends in natural disaster occurrence, DALYs rate due to IHD from 1990 to 2017 in 193 countries. **(A)** Trend in DALYs due to IHD, per 100,000 population per year. **(B)** Trend in the occurrence of natural disasters, per year. **(C)** Trend in total damage due to natural disasters, 1000 US$ per year.

The descriptive statistics of the natural disaster impact and DALYs rates due to IHD are summarized for 1990–2013, 2014–2017 and trends in the two periods separately (Table 1), with the average occurrence and casualty for subgroups of natural disasters (Figure 1). One-sample T-tests of the trends demonstrated a globally significant increase in natural disaster occurrences and decreases in DALYs rates between the two periods (p < 0.001). The correlations between natural disaster impacts and DALYs rates are summarized in Table 2. For all ages combined, the occurrence of natural disasters positively and significantly correlated with DALYs rates due to IHD for females and for both sexes (p < 0.05), and were marginally correlated for males, which can be visualized in maps of the trends (Figure 2). Geographically, the trends of the occurrence of DALYs shows an impressive consistency in heavily populated regions, including East Asia, Southeast Asia, South Asia, Western Europe and North America.

**Table 1:** Summary statistics of natural disaster impact, age-standardized mortality and DALYs due to IHD during two periods in 193 countries.

	1990 to 2013	2014 to 2017	Trends	P value*

Natural disaster				
Occurrence	1.77 ± 3.19	2.00 ± 3.56	0.23 ± 1.09	0.004
Casualty	3.21 × 10^4^ ± 2.86 × 10^5^	1.27 × 10^4^ ± 6.58 × 10^4^	–1.95 × 10^4^ ± 2.66 × 10^5^	0.310
Total damage	3.28 × 10^5^ ± 1.55 × 10^6^	7.70 × 10^5^ ± 4.59 × 10^6^	4.41 × 10^5^ ± 3.18 × 10^6^	0.055
DALYs				
Male	3030.950 ± 2154.273	2791.383 ± 2108.517	–239.567 ± 832.113	0.000
Female	1966.962 ± 1437.111	1743.577 ± 1388.023	–223.385 ± 502.153	0.000
Both	2494.384 ± 1757.926	2263.474 ± 1711.314	–230.91 ± 647.01	0.000

* One-sample T-test, test value = 0.

**Table 2:** Correlation of natural disaster impact, age-standardized mortality and DALYs due to IHD during two periods in 193 countries.

	Occurrence		Casualty		Total damage	

	R*	P value	R	P value	R	P value

Male	0.128	.076	–0.084	0.248	–0.032	0.660
Female	0.163	0.024	–0.105	0.145	–0.040	0.576
Total	0.146	0.043	–0.095	0.190	–0.036	0.620

* Pearson correlation.

The results of multivariate linear regression for males are shown in Table 3. In multivariate linear regression, the occurrence and total damage were independently and significantly associated with the DALYs rate (standardized β = 0.192 and 0.243, p < 0.05) after adjusting for confounding variables. In addition, significant correlations were found for variables, including tobacco, GDP per capita, life expectancy (males) and CO_2_ emissions. A considerable variance of 58.9% can be explained by such a model for males.

**Table 3:** Multivariate linear regression derived coefficients of natural disaster and socioeconomic variables on DALYs due to IHD for males, female and both sexes from 1990 to 2017 in 193 countries, weighted by population.

Factors	DALYs* for male			DALYs** for female			DALYs*** for both sexes		

	Coefficients (95% CI)	Standardized Coefficients	P value	Coefficients (95% CI)	Standardized Coefficients	P value	Coefficients (95% CI)	Standardized Coefficients	P value

Natural disaster									
Occurrence	39.955(7.473–72.436)	0.192	0.016	30.323(9.867–50.780)	0.230	0.004	31.122(5.547–57.698)	0.187	0.022
Casualty	NA^†^	0.088	0.462	NA^†^	0.027	0.823	NA^†^	0.154	0.216
Total damage	2.035 × 10^–5^(1.043 × 10^–5^–3.027 × 10^–5^)	0.243	0.000	1.103 × 10^–5^(4.952 × 10^–6^–1.712 × 10^–5^)	0.208	0.000	1.434 × 10^–7^(6.387 × 10^–6^–2.229 × 10^–5^)	0.214	0.000
Fat&Meat	NA	–0.050	0.464	NA	–0.013	0.832	NA	–0.057	0.390
Tobacco	27.361(8.214–46.509)	0.138	0.005	21.012(9.739–32.286)	0.167	0.000	23.758(9.124–38.392)	0.149	0.002
Alcohol	NA	0.037	0.511	NA	–0.068	0.114	NA	0.045	0.400
GDP per capita	–0.146(–0.174–0.119)	–0.642	0.000	–0.073(–0.089–0.057)	–0.508	0.000	–0.112(–0.133–0.090)	–0.612	0.000
Life expectancy (males)	–43.361(–77.655–90.67)	–0.125	0.013	NA	–0.083	0.098	–28.297(–54.091–2.503)	–0.107	0.032
Education expenditure (% of GDP)	NA	0.003	0.944	NA	–0.033	0.477	NA	–0.022	0.643
Urban population(% of total)	NA	0.135	0.116	12.961(1.605–24.316)	0.180	0.026	14.097(–0.667–28.860)	0.154	0.061
CO_2_ emissions	129.996(58.992–201.070)	0.276	0.000	67.230(19.899–114.562)	0.225	0.006	83.620(22.183–145.058)	0.221	0.008
Trade (% of GDP)	NA	0.022	0.654	NA	0.047	0.298	NA	0.038	0.415
Industry	NA	0.148	0.092	NA	0.128	0.118	NA	0.138	0.100

* Stepwise method was used, R^2^ = 0.589, F = 43.879, p = 0.000. ** Stepwise method was used, R^2^ = 0.643, F = 55.277, p = 0.000. *** Stepwise method was used, R^2^ = 0.628, F = 44.065, p = 0.000. ^†^ Not applicable for variables not included as predictors.

For females in Table 3, the occurrence and total damage were independently and significantly associated with the DALYs rate (standardized β = 0.230 and 0.208, p < 0.01) after adjusting for confounding variables. In addition, significant correlations were found for variables, including tobacco, GDP per capita, and CO_2_ emissions. A considerable variance of 64.3% can be explained by this model for females.

For both sexes in Table 3, occurrence and total damage were not surprisingly associated with the mortality rate (standardized β = 0.187 and 0.214, p < 0.01) after adjusting for confounding variables. Similarly, significant correlations were found for variables, including tobacco, GDP per capita, life expectancy (males), and CO_2_ emissions. A considerable variance of 62.8% can be explained by this model.

## Discussion

To the best of our knowledge, this is the first report with a positive association between trends in natural disasters and IHD with the metric of DALYs at the global level. Based on convincing evidence from 193 countries over a period of nearly 30 years, our study simultaneously demonstrates that both males and females are equally vulnerable to natural disasters. Although the worldwide trends of natural disasters and DALYs due to IHD vary significantly in opposite directions, our study provides a proof-of-concept demonstration that natural disasters should be taken into consideration as risk factors in relation to the burden of disease for IHD.

Our results are in accordance with a previous analysis demonstrating the impact of increasing IHD disease burden after the occurrence of a natural disaster. A systematic review including 26 studies of 12 earthquakes found that earthquakes may be associated with an increased incidence of acute coronary syndromes and cardiovascular mortality [3], Meanwhile, climate-change-related natural disasters, including biological, hydrological, meteorological and climatological disasters, cause negative effects on social and environmental determinants of health. A literature review [4] reported the lasted epidemiological evidence of the effects of climate changes on cardiovascular disease and concluded climate change adversely affects the cardiovascular system and cardiovascular diseases, especially in patients with a pre-existing heart disease. In addition, there is an accumulation of epidemiological and clinical evidence that all kinds of natural disasters, including volcanic eruptions, wildfires, dust storms, droughts, snowstorms and hurricanes, may negatively impact mortality and morbidity for cardiovascular diseases [10,11,12,13]. Recently, the guidelines for disaster medicine for patients with cardiovascular diseases by the Japanese Circulation Society JCS and JSH and JCC Joint Working Group [14] pointed out that natural disasters are associated with an increased risk of cardiovascular events, and it is believed that disaster-related cardiovascular events are a major intrinsic cause of death in a disaster.

In our study, the occurrence of a natural disaster positively and significantly correlated with DALYs rates due to IHD for females and both sexes, and were marginally correlated for males, even though both vary in opposite directions in the two periods over the nearly 30 years. Although the association between natural disasters and DALYs due to IHD is weak, the multivariate linear regression analysis suggested that each single incident significantly increase in the annual natural disaster occurrence rate was associated with an increase in DALYs of approximately 39.955 per 100,000 for males, 30.323 per 100,000 for females and 31.122 per 100,000 for both sexes. Our study showed that casualty caused by natural disasters was not significantly associated with DALYs. This finding suggests that the impact of natural disasters on IHD spreads far beyond the apparently directly affected population.

Although our study is consistent with previous studies on the negative effects of natural disasters on mortality and morbidity due to cardiovascular diseases, several advantages of our study merit mention. First, our study was performed to apply the DALY metric to quantifying morbidity and mortality for IHD, which has not been used by previous studies. Second, our study provided the first evidence at the global level from 193 countries over nearly 30 years, with extremely complete and reliable data. A series of socioeconomic variables were included as confounding variables in multivariate linear regression analyses, which enhanced the credibility of our results.

There are several explanations for the association between natural disasters and DALYs due to IHD. First, natural disasters lead to direct physical and chemical effects on health by extreme cold/hot temperatures or released substances, including particulate matter (PM), O_3_, elemental carbon (EC), organic carbon (OC), sulfates and nitrates. These can have direct effects such as autonomic system imbalances, hemodynamic disorders, dehydration, sympathetic and renin angiotensin system activation, oxidative stress and inflammation, a prothrombotic status, endothelial dysfunction and atherosclerosis [3,4,6]. Second, an increased incidence of post-traumatic stress disorder, depression, and other acute or chronic mental disorders have been observed after catastrophic death/injuries, environmental changes, infrastructure destruction, property loss, and even information dissemination following natural disasters [15,16,17]. Meanwhile, post-traumatic stress disorder and depression have been proven to be independently associated with an increased risk of mortality and morbidity for IHD. Third, natural disasters lead to an increased incidence of well-established risk factors for IHD, including hypertension, dyslipidemia, increased heart rate, BMI, smoking, alcohol abuse, high blood glucose, high-sodium diets and a lower socioeconomic level (such as GDP per capita) [3,18].

Besides the occurrence of a natural disaster, the multivariate linear regression analysis in our study suggested that total damage, tobacco and CO_2_ emissions are consistently and positively associated with DALYs due to IHD for males, females and both sexes. Considering the difficulty and inaccuracy of the evaluation of economic loss, measuring occurrences has an obvious advantage over total damage during a natural disaster. Tobacco consumption, an incontrovertible risk factor of CVD, is positively associated with DALYs due to IHD, which can be regarded as evidence of the reliability of our statistical methods. In addition, the significant association between CO_2_ emission and DALYs is another innovative finding of our study that had not been reported before and needs to be confirmed by additional studies. Meanwhile, our study also suggested that GDP per capita and life expectancy are consistently and inversely associated with DALYs due to IHD, which is consistent with previous studies [1,19].

In order to mitigate the public health crisis following a natural disaster, the main needs should be provided, which include water, food, shelter and medical interventions [20]. Furthermore, emerging guidelines or studies emphasizing in specialized interventions for noncommunicable diseases, such the guideline for disaster medicine for patients with cardiovascular diseases by the JCS.

Although the association between natural disasters and DALYs due to IHD is weak but statistically significant, the value of our study lies in the following points. First, it is important to increase awareness of the association between natural disasters and IHD. Second, although the occurrence of natural disasters might be a nonmodifiable risk factor for IHD, adaptation strategies, including environmental restoration, psychological interventions, lifestyle improvement and infrastructure reconstruction, can be developed and promoted for natural disaster responses and IHD disease burden reduction.

Despite using the best available data to explore the association between natural disasters and the disease burden for IHD, our study has several limitations. First, the main limitation of the ecological study design means that the results obtained from a population cannot be extrapolated on the individual level. Second, as shown in the inclusion criteria, the natural disasters included in our study were extremely destructive. Whether natural disasters with less severity have similar impacts on IHD is not clear. Third, our study reflects the long-term impact of disasters on IHD, with the short-term impact unclear. Fourth, the data on DALYs in our analysis is from the estimates obtained from the GBD using modeling. Finally, it is critical to determine the differences among the effects of natural disasters on specific clinical conditions of IHD (such as silent ischemia, stable angina or acute coronary syndrome) if the data are available.

## Conclusions

Our DALY metric analysis confirmed and quantified the long-term association between the occurrence of natural disasters and IHD at the global level. Meanwhile, a relevantly large percentage of the IHD burden can be aggravated by the occurrence of a natural disaster and the subsequent adverse effects. Our study indicates the necessity to develop and promote adaptation strategies for natural disaster responses and IHD disease burden reduction.

## Additional Files

The additional files for this article can be found as follows:

10.5334/gh.919.s1 File 1. Disaster 1990–2017.

10.5334/gh.919.s2 File 2. Final data.

10.5334/gh.919.s3 File 3. Analyses result.
